# A Case of Primary Refractory Immune Thrombocytopenia: Challenges in Choice of Therapies

**DOI:** 10.1155/2018/8207017

**Published:** 2018-07-03

**Authors:** Hanyin Wang, Hande Tuncer

**Affiliations:** ^1^Department of Internal Medicine, Tufts Medical Center, 800 Washington St., Boston, MA 02111, USA; ^2^Division of Hematology/Oncology, Tufts Medical Center, 800 Washington St, Boston, MA 02111, USA

## Abstract

The value of combination therapy for refractory ITP is not well defined. We present the case of a 29-year-old male with severe ITP refractory to initial standard therapy including steroids, IVIG, and subsequent splenectomy, who was treated with the combination therapy of rituximab, romiplostim, and mycophenolate and eventually developed thrombocytosis requiring plateletpheresis. Our case highlights the importance of the need to understand predictors of response to standard upfront treatment of acute ITP.

## 1. Introduction

Immune thrombocytopenia (ITP) is an immune-mediated disorder characterized by isolated thrombocytopenia with peripheral blood platelet count less than 100 × 10^9^/L [1]. An International Working Group (IWG) defined refractory ITP as cases that do not respond to or relapse after splenectomy while require treatment to reduce the risk of clinically significant bleeding [1]. Despite advances in the treatment of ITP in the past two decades, management of refractory ITP remains to be a challenge. The optimal sequence of treatment options is unknown. Furthermore, the role of combination therapy is not well defined.

We present the case of a 29-year-old male with pituitary apoplexy and severe ITP refractory to initial standard treatment including steroids and IVIG requiring further management including splenectomy, rituximab, romiplostim, and mycophenolate.

## 2. Case Presentation

A 29-year-old man with no significant past medical history presented to emergency department with 2 days of epistaxis and petechiae. The patient experienced upper respiratory infection symptoms 5 days prior to presentation. He was not taking any home medication. His family history was not significant. His vital signs were stable. Physical exam was notable for oral blisters and petechial rash over extremities. He was found to have a platelet count of 1 × 10^9^/L. The rest of CBC was normal. Peripheral blood smear confirmed profound thrombocytopenia with normal platelet size and no platelet clumping. TSH, hepatitis C antibody, HIV antibody, *H. pylori* stool antigen, CMV PCR, and EBV PCR were all negative. Coagulation function and ADAMTS13 activity were normal. The respiratory viral panel was positive for rhinovirus. Bone marrow biopsy showed trilineage maturing hematopoiesis with markedly increased megakaryocytes. Bone marrow flow cytometry and cytogenetic analysis were unremarkable. He was diagnosed of ITP possibly triggered by rhinovirus infection.

**Figure 1:**
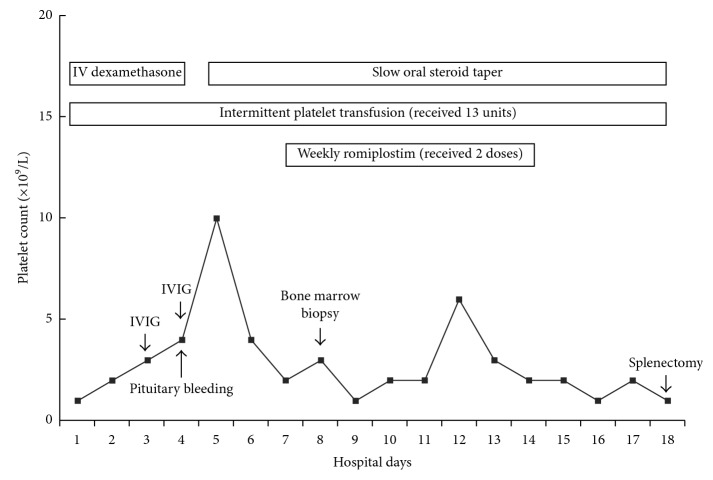
Platelet counts before splenectomy.

After admission, the patient was immediately started on IV dexamethasone 40 mg daily for 4 days. IVIG 1 g/kg/day was administered on hospital days 4 and 5. The patient developed severe headache on hospital day 5. Head CT followed by sella MRI demonstrated a small focus of hemorrhage into a pituitary macroadenoma consistent with pituitary apoplexy. At this time, he was started on intravenous aminocaproic acid. He received daily platelet transfusion with no response in platelet count (Figure 1). Romiplostim was administered on hospital day 7 (6.5 *μ*g/kg) and day 14 (10 *μ*g/kg). Anti-D could not be used given his Rh-negative blood type. Dexamethasone was transitioned to prednisone 1 mg/kg/day and then gradually tapered down. His platelet count was refractory to all treatments above and remained at a single-digit level. Eventually, he underwent uncomplicated laparoscopic splenectomy on hospital day 18. He was discharged on postoperative day 2 with the maintenance dose of corticosteroids as his platelet count rose to 215 × 10^9^/L.

**Figure 2:**
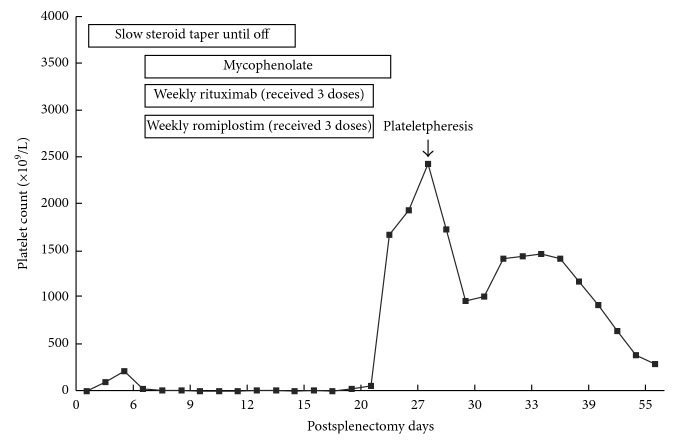
Platelet counts after splenectomy.

On postoperative day 7, he was found to have a platelet count of 8 × 10^9^/L during clinic follow-up. He was subsequently started on rituximab 375 mg/m^2^ weekly, romiplostim 10 *μ*g/kg weekly, and mycophenolate 500 mg twice daily. On postoperative day 20, his platelet count increased to 60 × 10^9^/L. On postoperative day 27, he developed a mild headache, and his platelet count was found to be 2424 × 10^9^/L (Figure 2). A head CT was done which showed no acute process but findings compatible with resolving pituitary apoplexy. He was admitted to hospital for urgent plateletpheresis given extreme thrombocytosis (>1000 × 10^9^/L) associated with headache and started on aspirin 81 mg q.d. for thromboprophylaxis. The plateletpheresis was performed with whole blood daily for three consecutive days. Platelet count immediately after last plateletpheresis was 1012 × 10^9^/L. Platelet count remained elevated in the 1000–1400 × 10^9^/L range for the first week after discharge before it started to normalize (Figure 2). All ITP treatments were discontinued. Platelet count was 288 × 10^9^/L at postoperative week 8 and 477 × 10^9^/L at postoperative week 39.

## 3. Discussion

There is little evidence to guide a sequence of treatments for patients with refractory ITP after an initial treatment course with corticosteroids (or IVIG or anti-D). Evolving therapies in the past 10 years, especially thrombopoietin receptor agonists (TPO-RAs) and rituximab, have decreased the rate of splenectomy as second-line treatment [2]. IWG recommended to defer splenectomy until the chronic phase of ITP (>6 months after diagnosis) in most cases [3]. However, splenectomy remains the treatment modality with the best response rates and the lower incidence of relapse [2]. In our case, splenectomy was adopted early because the patient developed critical pituitary apoplexy and had failed corticosteroids, IVIG, and romiplostim.

Compared to sequence single-agent therapy, the rationale for combination therapy is to target different pathways involved in platelet destruction with minimal overlapping toxicities. In our case, mycophenolate was added concurrently with romiplostim and rituximab when we failed to see an expected treatment response after splenectomy, and the platelet count again dropped to a critical level. Furthermore, the patient already failed romiplostim in the prior hospital course. The value of combination therapy for refractory ITP was not well defined. Immunosuppression therapies have been successfully added to rituximab [4] and TPO-RAs [5, 6], respectively. Since rituximab has a relatively long time to response (Table 1), TPO-RAs have been suggested as rational agents to combine with rituximab to induce a rapid increase in platelet counts. Based on the different mechanism of action of rituximab (reduces platelet destruction by depleting B cells) and TPO-RAs (stimulate platelet production in the bone marrow), synergistic effects of such a combination can be more than additive [7, 8]. Zhou et al. compared rituximab plus recombinant human thrombopoietin with rituximab alone for corticosteroid-resistant or relapsed ITP [9]. The combination group in this study has a significantly higher complete response rate and shorter time to response; however, there is no difference in long-term response [9].

The extreme thrombocytosis in our case appeared to be associated with combination therapy for ITP in the postsplenectomy setting, as discontinuation of ITP medications led to gradual improvement in platelet count. The most effective component(s) of our regimen that led to extreme thrombocytosis cannot be ascertained. Our patient developed thrombocytosis 3 weeks after initiation of the combination therapy. Based on the time to response (Table 1), both rituximab and romiplostim should have started to show effect, but mycophenolate has not reached its time to response. Although postsplenectomy reactive thrombocytosis has an incidence rate of 75% [11, 12], it could not explain our patient's drop of platelet counts in the first week postoperatively. Thrombocytosis has been associated with both rituximab [13, 14] and romiplostim [15, 16] as single therapy, but in these cases, thrombocytosis never required plateletpheresis. In the largest trial assessing rituximab plus recombinant human thrombopoietin, the incidence of thrombocytosis was not reported, but the incidence of thrombosis was only 1.3% [9]. It is possible that the postsplenectomy status made our patient more susceptible to thrombocytosis. Severe postsplenectomy thrombocytosis requiring plateletpheresis has been reported in patients receiving romiplostim preoperatively [17, 18], but unlike our case, these patients suffered from thrombocytosis in the immediate postoperative period. When rituximab and romiplostim are used as single therapy, splenectomy does not influence their response rate [13, 19]. Although doses of the three medications in our case were well within the recommended range, it would be interesting to know if lower doses in the setting of combination therapy would decrease the rate of thrombocytosis.

In conclusion, we describe an unusual and challenging case of primary refractory ITP. The case highlights the need to understand predictors of response and relapse in this particular setting. Furthermore, the optimal choice and sequence of therapies remains poorly defined. Although there is some successful experience of adding rituximab to TPO-RAs, there is an inherit risk of thrombocytosis with possible synergistic effects from such a combination. The concurrent use of rituximab, mycophenolate, and TPO-RAs in the postsplenectomy period may further augment thrombocytosis risk in selected patients. Future studies are warranted to understand the predictors of response and to evaluate the safety and efficacy of combining rituximab, TPO-RAs, and immunosuppression therapy, especially in the postsplenectomy setting for patients with refractory ITP.

## Figures and Tables

**Table 1:** Time to response of selected treatment options for adults with ITP [3, 10].

Treatment type	Initial response (days)	Peak response (days)
Splenectomy	1–56	7–56
Rituximab	7–56	14–180
Romiplostim	5–14	14–60
Mycophenolate mofetil	28–42	N/A
